# Long-term safety and decrease of pill burden by tenapanor therapy: a phase 3 open-label study in hemodialysis patients with hyperphosphatemia

**DOI:** 10.1038/s41598-023-45080-9

**Published:** 2023-11-04

**Authors:** Fumihiko Koiwa, Yu Sato, Meiko Ohara, Kaoru Nakanishi, Masafumi Fukagawa, Tadao Akizawa

**Affiliations:** 1 Division of Nephrology, Department of Internal Medicine, Showa University Fujigaoka Hospital, 1-30 Fujigaoka, Aoba-Ku, Yokohama, 227-8501 Japan; 2 R&D Division, Kyowa Kirin Co., Ltd., Tokyo, Japan; 3 Division of Nephrology, Endocrinology, and Metabolism, Department of Internal Medicine, Tokai University School of Medicine, Kanagawa, Japan; 4 Division of Nephrology, Department of Medicine, Showa University School of Medicine, Tokyo, Japan

## Abstract

Phosphate binders (PBs) generally have a high pill burden. Tenapanor selectively inhibits sodium/hydrogen exchanger isoform 3, reducing intestinal phosphate absorption. Tenapanor is a novel drug administered as a small tablet, twice daily. This multicenter, open-label, single-arm, phase 3 study aimed to evaluate the long-term safety of tenapanor and its efficacy in decreasing PB pill burden. Tenapanor 5 mg twice daily was administered to hemodialysis patients with serum phosphorus level 3.5–7.0 mg/dl at baseline; the dose could be increased up to 30 mg twice daily. Patients could also switch from PBs. The primary endpoint was safety during 52-week administration. The key secondary endpoint was a ≥ 30% reduction in the total pill number of daily PBs and tenapanor from baseline. Of 212 patients starting treatment, 154 completed the study. Diarrhea was the most frequent adverse event, occurring in 135 patients (63.7%); most events were classified as mild (74.8%). No clinically significant changes occurred other than serum phosphorus level. At Week 52/discontinuation, 158/204 patients (77.5%) achieved the key secondary endpoint. Complete switching from PBs to tenapanor was achieved in 50–76 patients (26.7%–41.5%), and 80 patients (51.9%) at Week 8–12 and Week 50, respectively. Serum phosphorus remained generally stable within the target range (3.5–6.0 mg/dl). These findings suggest the long-term safety and tolerability of tenapanor. Tenapanor could reduce or eliminate PB pill burden while controlling serum phosphorus levels.

*Trial registration*: NCT04771780

## Introduction

Phosphorus accumulation in patients undergoing hemodialysis may lead to hyperphosphatemia and calcium phosphate deposits in the vascular walls, heart, and kidneys, resulting in calcification and increasing the risk of cardiovascular morbidity and mortality^1^. Dialysis and dietary intervention are important in managing phosphorus levels. However, removing phosphorus by dialysis has limitations, and the strict dietary restrictions (e.g., low daily dietary protein intake) may cause malnutrition and increased mortality risk^2^. For these reasons, the adequate management of serum phosphorus levels by dialysis and dietary intervention is often difficult; thus, administration of phosphate binders is essential^3^. Phosphate binders are effective in treating hyperphosphatemia; however, some disadvantages raise concerns, including hypercalcemia, gastrointestinal symptoms, and accumulation of metals in organs^4^.

In addition, an important issue in patients receiving hemodialysis is the phosphate binder pill burden. Phosphate binders have large pill sizes and account for many of the pills hemodialysis patients receive, needing to be taken three times daily before or after each meal. Hence, the oral administration of phosphate binders causes a high pill burden^5^, which has a detrimental effect on drug adherence, resulting in inadequate phosphorus management. Moreover, water intake is restricted in patients receiving hemodialysis, making it difficult for some patients to adhere to oral treatment regimens^6,7^. There is, therefore, a pressing need for treatments with more manageable pill burdens to control serum phosphorus levels.

Tenapanor is a novel drug that reduces intestinal phosphate absorption via the selective inhibition of sodium/hydrogen exchanger isoform 3 (NHE3)^8^. NHE3 plays an important role in sodium absorption from the gastrointestinal tract. Its inhibition results in the decreased permeability of epithelial cell junctions, thereby reducing paracellular phosphate influx. This leads to decreased serum phosphorus levels, and the simultaneous accumulation of sodium in the intestinal tract causes loose stools. Additionally, it is plausible that tenapanor could contribute to decreasing the phosphate binder pill burden, or eliminate the need for phosphate binder pills, because the dose consists of one tablet that is smaller than conventional phosphate binders and is taken twice daily.

A Japanese phase 3 study of tenapanor in patients with hyperphosphatemia undergoing hemodialysis determined that tenapanor significantly reduced serum phosphorus levels compared with placebo and was well tolerated during 8-week administration^9^. However, the safety of long-term tenapanor administration has not yet been evaluated in Japanese patients receiving hemodialysis. Furthermore, its long-term efficacy in decreasing phosphate binder pill burden has not yet been confirmed. Therefore, this study aimed to simultaneously evaluate the long-term safety of tenapanor and its effect on pill burden decrease.

## Methods

### Study design

**Figure 1:**
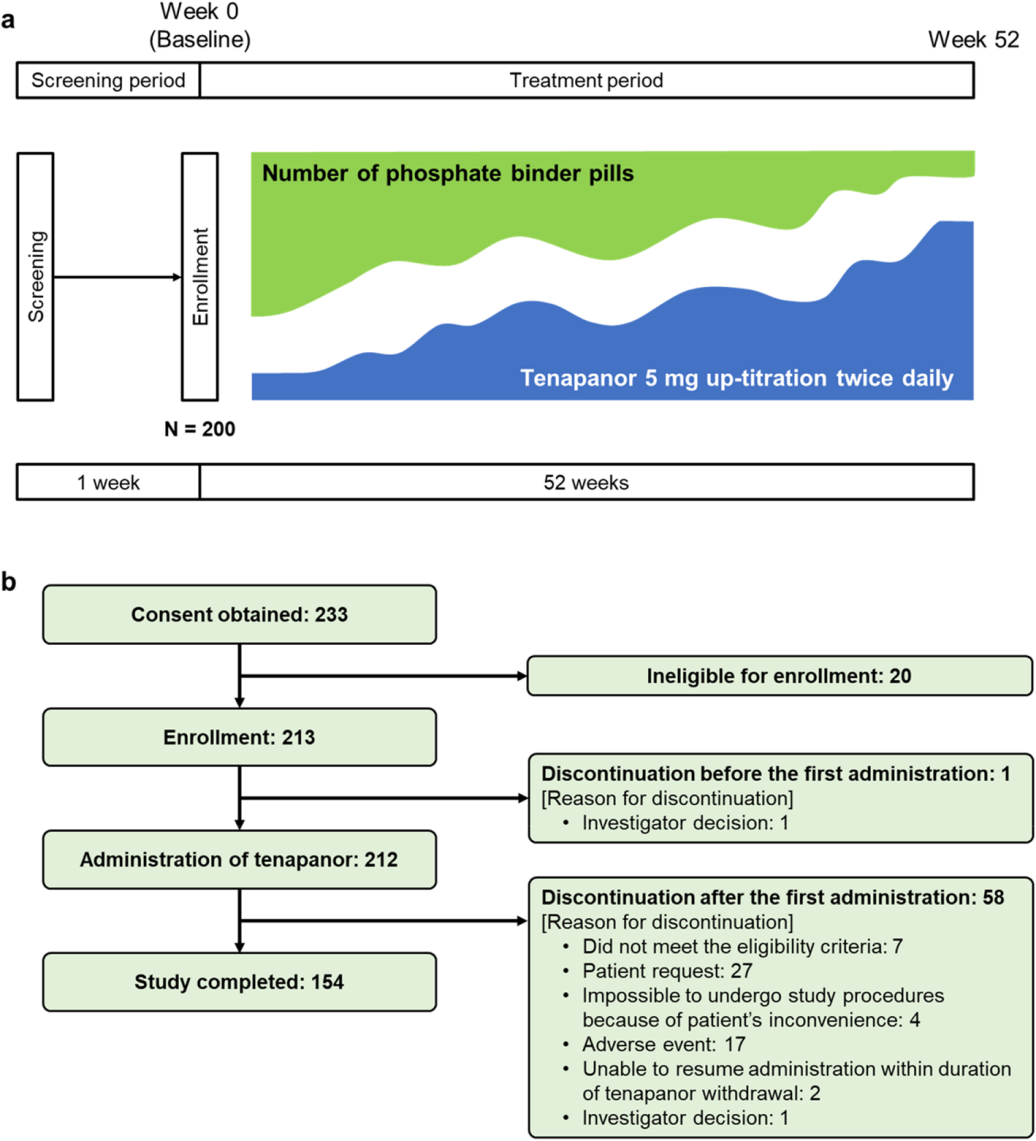
(**a**) Study design; (**b**) patient disposition.

This study was a multicenter, open-label, single-arm, long-term phosphate binder switching, phase 3 study conducted at 30 sites in Japan from March 2021 to June 2022. The institutional review board at each participating site (Sapporo Medical Association's Institutional Review Board, Review Board of Human Rights and Ethics for Clinical Studies Institutional Review Board, Yokohama Minoru Clinic Institutional Review Board, Adachi Kyosai Hospital Institutional Review Board, and Jinbo Orthopedic Surgery Institutional Review Board) approved the study protocol and related documents. The list of participating centers is shown in Supplementary Text S1. This study adhered to Good Clinical Practice guidelines, the Declaration of Helsinki, and local laws and regulations. All patients provided written informed consent to participate. The study was registered on 25 February 2021 at ClinicalTrials.gov under the identifier NCT04771780. The study period comprised a 1-week screening period followed by a 52-week treatment period (Fig. 1a).

### Participants

The main inclusion criteria were as follows: patients aged ≥ 20 years; undergoing hemodialysis three times weekly for at least 12 weeks before screening; taking the same dosage of phosphate binders for 4 weeks before screening; having serum phosphorus level of ≥ 3.5 mg/dl and ≤ 7.0 mg/dl at screening; receiving vitamin D therapy, calcimimetics, bisphosphonates, calcitonin, selective estrogen receptor modulators, or teriparatide, if any, at an unchanged dosage for 4 weeks before screening; and having Kt/V urea of ≥ 1.2 in the most recent routine medical examination before screening.

The main exclusion criteria were as follows: intact parathyroid hormone > 600 pg/ml at screening; a history of inflammatory bowel disease or diarrhea-predominant irritable bowel syndrome; or the presence of diarrhea or loose stools, defined as Bristol Stool Form Scale score ≥ 6 and three or more defecations per day for ≥ 2 days, between screening and enrollment.

### Treatment

Following the screening period, patients were administered tenapanor orally at a starting dose of 5 mg twice daily, immediately before meals (breakfast and dinner) for 52 weeks in addition to phosphate binders at the dose administered at screening. The tenapanor dose could be adjusted in a stepwise manner from 5 mg to 10, 20, and 30 mg twice daily. The dose of tenapanor and phosphate binders was adjusted based on the most recent serum phosphorus level (Supplementary Fig. S1), while controlling serum phosphorus levels and switching from phosphate binders to tenapanor. Patients were categorized by baseline serum phosphorus levels of ≤ 6.0 mg/dl (control target: baseline range of ± 0.5 mg/dl) and ≥ 6.1 mg/dl (control target: ≥ 5.5 mg/dl and ≤ 6.0 mg/dl). The tenapanor dose was increased and the phosphate binder dose was reduced in principle. If the most recent serum phosphorus level was < 3.5 mg/dl or if an adverse event (AE) occurred, then tenapanor and phosphate binders could be reduced or withdrawn.

Aluminium preparations, niceritrol, nicotinamide, colestimide, cholestyramine, other supplements for phosphorus adsorption, anti-receptor activator of nuclear factor kappa-Β ligand antibody preparations, anti-sclerostin antibody preparations, and peritoneal dialysis were prohibited. Furthermore, the additional use or dose modification of drugs related to mineral metabolism was restricted, and changes in hemodialysis conditions were permitted when deemed necessary for the safety of patients.

### Data collection

Data were collected from electronic case report forms. Paper patient diaries were used to collect medication adherence and defecation status data. In the study periods, samples for clinical laboratory tests were collected before or after dialysis, and pre-dialysis samples were obtained right before the first dialysis session of the week. Baseline samples were collected on Day 1 (Week 0) before starting treatment. The parameters at each time point were assessed at a central laboratory.

### Study endpoints

The primary endpoint was the safety evaluation regarding AEs, changes in laboratory values, vital signs, and standard 12-lead electrocardiograms. Changes in Bristol Stool Form Scale score and stool frequency were also assessed.

The key secondary endpoint was the achievement of a ≥ 30% reduction in the total pill number of phosphate binders and tenapanor prescribed daily in the last 3 weeks before Week 52 or the time of discontinuation compared with the pill number of phosphate binders prescribed daily at baseline.

Secondary endpoints included the following: the total daily pill number, weight, and volume of phosphate binders and tenapanor at each time point; achievement of a ≥ 30% decrease from the pill number of phosphate binders and tenapanor prescribed daily at each time point compared with the pill number of phosphate binders at baseline; their changes and percentage from baseline; and serum phosphorus levels and changes from baseline at each time point.

### Statistical analysis

Categorical data were summarized as the frequency and percentage, and continuous data were summarized using the number of patients, mean, standard deviation (SD), minimum, median, and maximum. The safety analysis set comprised all patients who received tenapanor. For efficacy, the primary analysis set comprised the modified intention-to-treat population, defined as all patients who received tenapanor and whose serum phosphorus level was measured after the start of tenapanor treatment.

For the primary endpoint, the incidences of all AEs and drug-related AEs that occurred or worsened after the start of tenapanor treatment were summarized using Medical Dictionary for Regulatory Activities preferred term and system organ class. Laboratory parameters and vital signs were summarized using summary statistics at each time point. The percentage of patients who achieved the key secondary endpoint was calculated, along with 95% confidence intervals (CIs). The analysis for the key secondary endpoint was performed against the threshold level of 20% and a one-sided significance level of 1.25% at the data cut-off point and final point of the treatment period. Statistical analyses were conducted using SAS version 9.4 (SAS Institute, Tokyo, Japan).

The sample size was based on the International Council for Harmonisation of Technical Requirements for Pharmaceuticals for Human Use guidelines^10^. As a reference, a minimum of 100 patients was required for a 52-week study evaluating the long-term safety of tenapanor. Based on previous phase 2 and 3 studies^11,12^, we assumed a withdrawal rate of 50%. Thus, the target sample size was set as 200 patients.

## Results

### Patient disposition and background characteristics

A total of 233 patients provided informed consent, and 213 patients were eligible for enrollment. Tenapanor administration was started in 212 patients. In total, 58 patients discontinued treatment, and 154 patients completed the study up to Week 52 (Fig. 1b). The main reasons for discontinuation were patient request (n = 27) and AEs (n = 17). The primary analysis set included 204 patients, and the safety analysis set included 212 patients. Of 204 patients, more than half were male (61.3%), and the mean (SD) age was 63.4 (10.45) years. Chronic glomerulonephritis (36.3%) and diabetic nephropathy (29.9%) were the most common primary diseases (Table 1). At baseline, the mean (SD) daily pill number of phosphate binders prescribed was 11.4 (7.6). The most common types of phosphate binders used at baseline were lanthanum carbonate (49.5%) and calcium carbonate (47.1%). At baseline, 152 patients (74.5%) used two or more types of phosphate binders, while 52 (25.5%) used one type. Mean (SD) baseline serum phosphorus level was 5.24 (0.96) mg/dl.

**Table 1:** Patient demographics in the primary analysis set.

Patient characteristic		N = 204
Sex [n (%)]	Female	79 (38.7)
	Male	125 (61.3)
Age, years (mean ± SD)		63.4 ± 10.5
Body weight, kg (mean ± SD)		62.9 ± 14.0
Type of dialysis [n (%)]	Hemodialysis	66 (32.4)
	Hemodiafiltration	136 (66.7)
	Other	2 (1.0)
Dialysate calcium concentration, mEq/l [n (%)]	2.5	34 (16.7)
	2.75	81 (39.7)
	3.0	62 (30.4)
	Other	27 (13.2)
Kt/V value (mean ± SD)		1.64 ± 0.32
nPCR, g/kg/day (mean ± SD)		0.91 ± 0.15
Primary disease [n (%)]	Diabetic nephropathy	61 (29.9)
	Chronic glomerulonephritis	74 (36.3)
	Nephrosclerosis	19 (9.3)
	Polycystic kidney disease	14 (6.9)
	Chronic pyelonephritis	3 (1.5)
	Other	33 (16.2)
Type of phosphate binder taken at baseline [n (%)]	Bixalomer	38 (18.6)
	Sevelamer	62 (30.4)
	Ferric citrate	85 (41.7)
	Calcium carbonate	96 (47.1)
	Lanthanum carbonate	101 (49.5)
	Sucroferric oxyhydroxide	25 (12.3)
Number of phosphate binder types taken at baseline [n (%)]	One type	52 (25.5)
	Two or more types	152 (74.5)
Pill number of phosphate binders per day at baseline, pills (mean ± SD)		11.4 ± 7.6
Total daily weight of phosphate binders at baseline, mg (mean ± SD)		5,730.8 ± 3,539.2
Total daily volume of phosphate binders at baseline, mm^3^ (mean ± SD)		4,590.2 ± 3,525.1
Serum phosphorus level at baseline, mg/dl (mean ± SD)		5.24 ± 0.96
Bristol Stool Form Scale score at baseline (mean ± SD)		3.8 ± 1.1
Stool frequency at baseline, times/week (mean ± SD)		7.4 ± 3.7

*Kt/V* fractional urea clearance, *nPCR* normalized protein catabolic rate, *SD* standard deviation.

### Primary endpoints

AEs and drug-related AEs with an incidence ≥ 5% are described in Table 2. AEs and drug-related AEs occurred in 204 patients (96.2%) and 134 patients (63.2%), respectively. One patient died during the study period because of COVID-19, with no causal relationship to the study drug. The AE of diarrhea occurred in 135 patients (63.7%), in whom the severity was classified as mild, moderate, and severe in 74.8%, 23.7% and 1.5%, respectively. The first onset of drug-related diarrhea was mostly observed by Week 3; thereafter, there were few new occurrences of diarrhea during the continuous 52-week administration (Supplementary Fig. S2). Of the 17 patients (8.0%) who discontinued the study drug because of AEs, nine patients (4.2%) discontinued because of diarrhea. Sixty-two serious AEs were observed in the study. Of those serious AEs, serious drug-related AEs occurred in three patients (1.4%). These were diverticular perforation, gastric ulcer hemorrhage, and colitis.

**Table 2:** AEs and drug-related AEs with an incidence ≥ 5%.

		N = 212
		n (%)
**AEs**		**204 (96.2)**
Diarrhea		135 (63.7)
Vaccination complication		42 (19.8)
Pyrexia		37 (17.5)
Contusion		28 (13.2)
Nasopharyngitis		20 (9.4)
Back pain		20 (9.4)
Shunt stenosis		18 (8.5)
Hyperkaliemia		15 (7.1)
Arthralgia		13 (6.1)
COVID-19		12 (5.7)
Pain in extremity		12 (5.7)
Nausea		11 (5.2)
Ligament sprain		11 (5.2)
Shunt occlusion		11 (5.2)
Muscle spasms		11 (5.2)
Headache		11 (5.2)
Severity of diarrhea^a^	Mild	101 (74.8)
	Moderate	32 (23.7)
	Severe	2 (1.5)
Discontinuation	AEs (diarrhea)	9 (4.2)
**Drug-related AEs**		**134 (63.2)**
Diarrhea		120 (56.6)
**Serious AEs**		**44 (20.8)**
Death		1 (0.5)
Other serious AEs		43 (20.3)
**Serious drug-related AEs**		**3 (1.4)**
Death		0 (0.0)
Other serious drug-related AEs		3 (1.4)

^a^Severity was determined by the attending investigator according to the following definitions: mild, signs or symptoms present but not interfering with daily activities; moderate, interferes with daily activities due to discomfort or affects clinical status; and severe, inability to engage in daily activities or significant impact on clinical status.

*AE* adverse event.

Table 3 shows laboratory values at Weeks 0, 8, 24, 40, and 52. A decrease in bicarbonate levels was observed. Other than changes in serum phosphorus levels, no clinically significant changes were observed in laboratory values after 52-week administration of tenapanor. Serum albumin and normalized protein catabolic rate, as indices of nutritional status, did not change throughout the study, and there were no clinically relevant alterations in vital signs or electrocardiograms.

**Table 3:** Laboratory values.

	Week					Change from Baseline to Week 52
	0	8	24	40	52	
Serum phosphorus, mg/dl	5.26 (1.00)	4.77 (1.07)	5.15 (1.07)	5.12 (1.08)	5.11 (1.17)	− 0.08 (1.37)
Corrected serum calcium, mg/dl	9.03 (0.52)	8.98 (0.49)	8.86 (0.52)	8.87 (0.55)	8.86 (0.59)	− 0.14 (0.61)
Calcium × phosphorus product, mg/dl^2^	47.22 (9.01)	42.79 (9.88)	45.59 (9.90)	45.46 (10.25)	45.26 (10.75)	− 1.39 (12.67)
Intact PTH, pg/ml^a^	126.5 (14, 527)	134.0 (13, 584)	144.0 (14, 506)	139.0 (12, 636)	132.5 (9, 587)	15.5 (− 294, 447)
Intact FGF23, pg/ml^a^	2,225.0 (41.0, 38,300.0)	1,340.0 (33.2, 28,400.0)	1,640.0 (28.3, 63,700.0)	1,230.0 (23.9, 45,500.0)	950.5 (25.1, 56,900.0)	− 571.7 (− 26,200.0, 28,200.0)
Sodium, mEq/l	139.3 (2.47)	138.9 (2.61)	138.8 (2.75)	139.2 (2.78)	139.1 (2.52)	− 0.2 (2.40)
Potassium, mEq/l	4.91 (0.62)	5.04 (0.76)	4.92 (0.73)	5.02 (0.76)	4.90 (0.71)	0.04 (0.65)
Chloride, mEq/l	103.2 (3.38)	103.7 (3.54)	103.3 (3.40)	103.6 (3.18)	103.5 (3.21)	0.5 (2.97)
Magnesium, mg/dl	2.53 (0.33)	2.50 (0.30)	2.40 (0.34)	2.44 (0.33)	2.38 (0.32)	− 0.16 (0.30)
Bicarbonate, mmol/l	19.12 (2.19)	18.26 (2.32)	18.31 (2.37)	17.58 (2.31)	17.25 (2.45)	− 1.96 (2.32)
Alkaline phosphatase, U/l	77.6 (29.9)	79.9 (28.5)	83.2 (31.9)	85.8 (29.8)	86.6 (33.3)	11.3 (28.7)
Bone-specific alkaline phosphatase, µg/l	14.10 (6.59)	15.38 (7.48)	16.94 (9.50)	18.35 (9.55)	18.71 (10.75)	4.57 (9.10)
Albumin, g/dl	3.60 (0.30)	3.57 (0.28)	3.60 (0.27)	3.62 (0.27)	3.61 (0.28)	− 0.01 (0.23)
nPCR, g/kg/day	0.91 (0.15)	0.91 (0.16)	0.91 (0.16)	0.92 (0.17)	0.89 (0.17)	− 0.02 (0.12)

All values are mean (SD) unless stated otherwise.

^a^Median (min, max).

Calcium × phosphorus product, intact PTH, and intact FGF23 were analyzed in the primary analysis set (N = 204), and other parameters were analyzed in the safety analysis set (N = 212).

*PTH* parathyroid hormone, *FGF23* fibroblast growth factor23, *nPCR* normalized protein catabolic rate, *SD* standard deviation.

**Figure 2:**
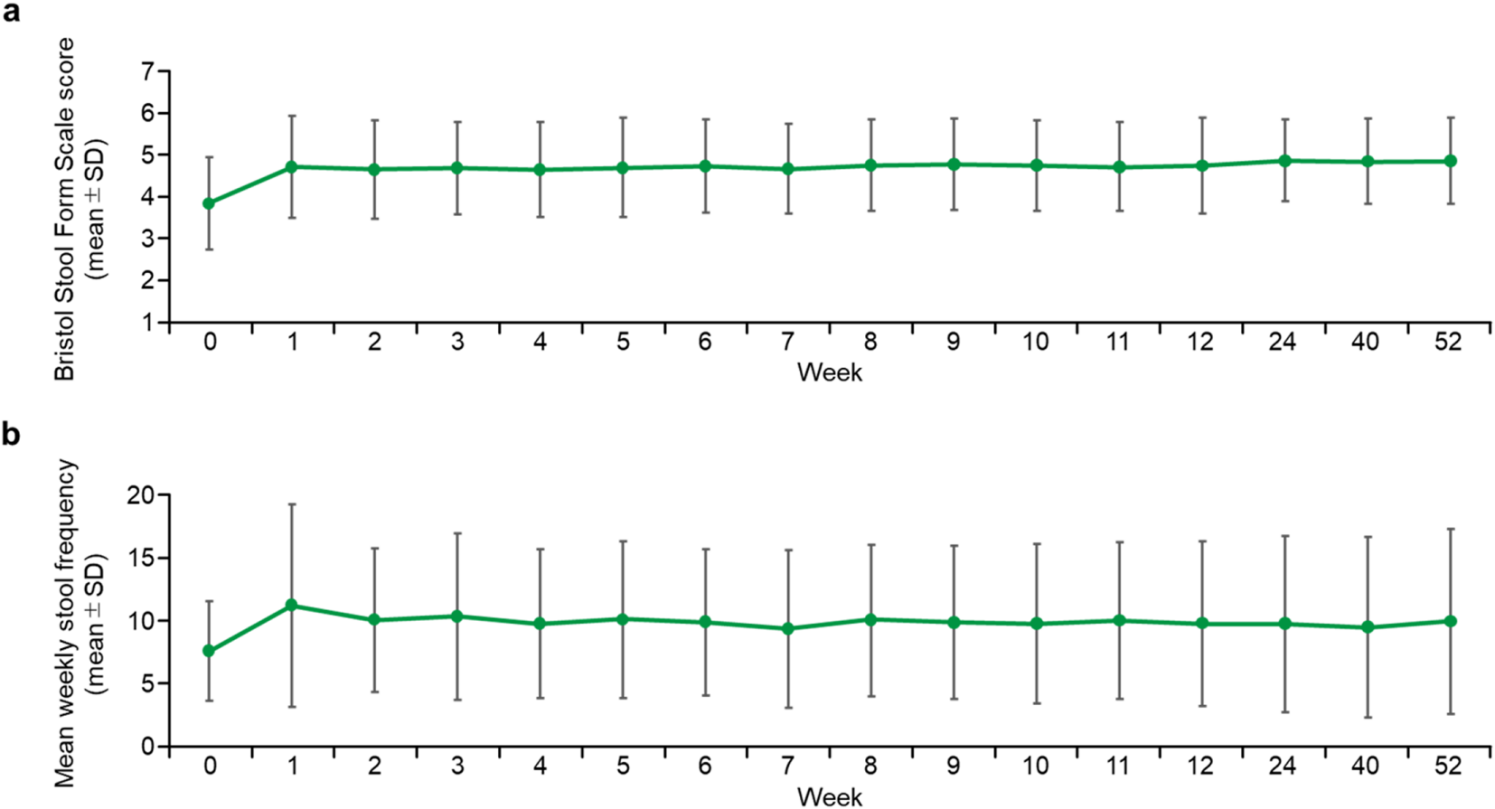
(**a**) Changes in mean Bristol Stool Form Scale score; (**b**) changes in mean weekly stool frequency. SD, standard deviation.

Mean Bristol Stool Form Scale score increased after the start of tenapanor treatment and remained stable after Week 2 (Fig. 2a). Mean (SD) Bristol Stool Form Scale scores were 3.8 (1.1) at Week 0 and 4.8 (1.0) at Week 52. Weekly stool frequency increased at Week 1 and remained stable thereafter (Fig. 2b). Mean (SD) weekly frequencies were 7.6 (4.0) at Week 0 and 9.9 (7.4) at Week 52.

### Secondary endpoints

**Figure 3:**
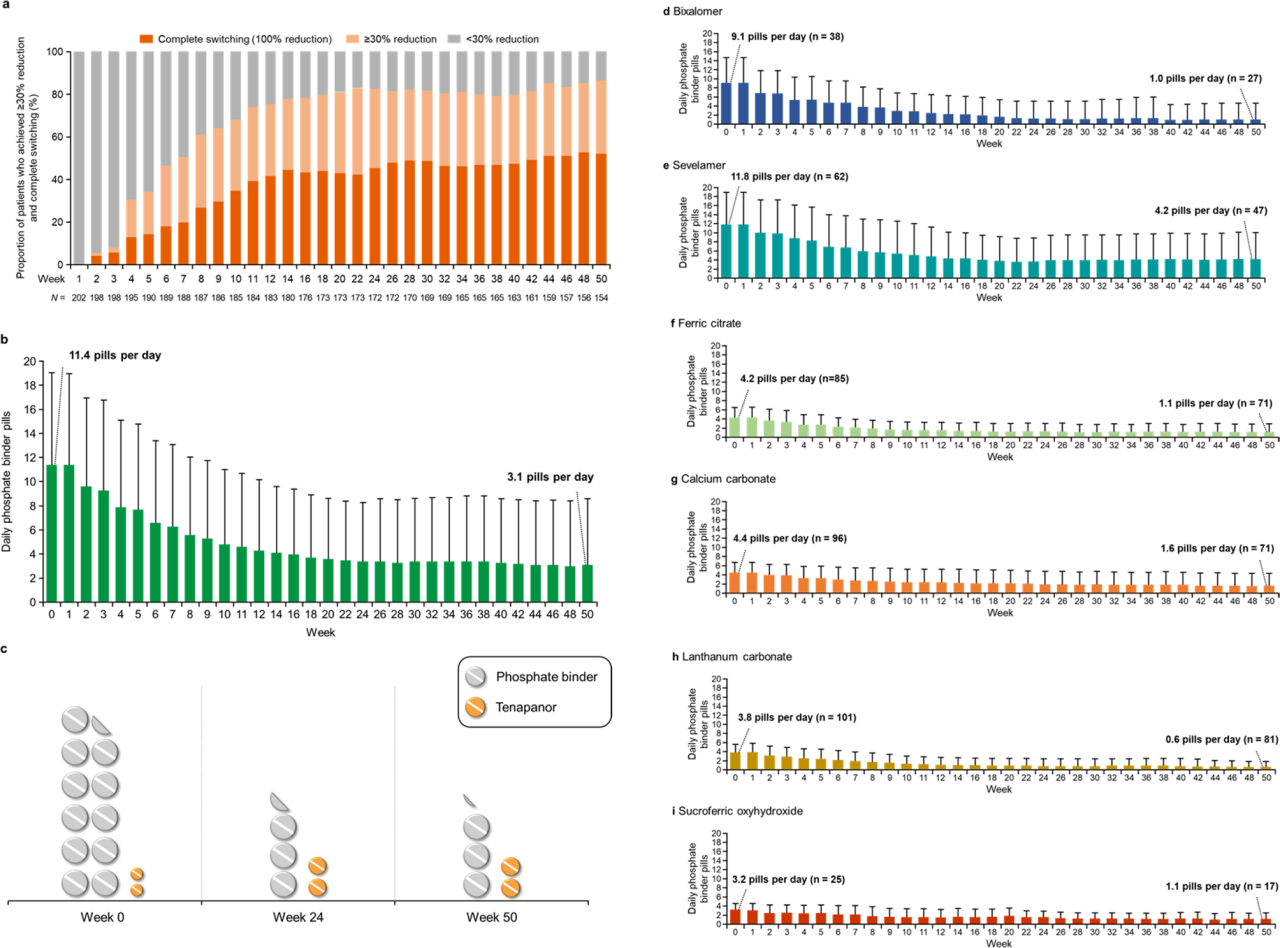
(**a**) Proportion of patients who achieved ≥ 30% reduction in the total number of pills and complete switching from phosphate binders to tenapanor; (**b**) changes in the mean pill number of phosphate binders prescribed per day; (**c**) image showing the decrease in the mean number of daily pills; (**d**)**–**(**i**) changes in the mean number of prescribed pills per day by type of phosphate binder. In panel (**c**), the number of pills indicates the mean daily number of pills taken, and the size is proportional to the cube root of the mean volume of the daily pills.

At Week 52, or the time of discontinuation, the number of patients achieving a ≥ 30% reduction in the total pill number of phosphate binders and tenapanor per day (the key secondary endpoint) was 158/204 (77.5%; 95% CI: 71.1, 83.0; P < 0.0001) (Table 4). Complete switching from phosphate binders to tenapanor (100% decrease of phosphate binders) was achieved in 93 patients (45.6%; 95% CI: 38.6, 52.7). The achievement of a reduction in pill number and complete switching categorized by the number of phosphate binder types at baseline are described in Table 4. Among patients who received multiple types of phosphate binders at baseline, the number of patients achieving a ≥ 30% reduction was 120/152 (78.9%; 95% CI: 71.6, 85.1). Figure 3a shows the time course of the achievement of phosphate binder pill reduction (≥ 30% reduction and complete switching). By Week 7, more than half of the patients had achieved a ≥ 30% reduction in the total number of pills taken, with the proportion of patients continuing to increase thereafter. Eighty patients (44.4%) achieved complete switching by Week 14, and the proportion remained generally unchanged after Week 14.

**Table 4:** Achievement of reduction in pill number and complete switching in the most recent 3 weeks at the end of the study.

	Number of phosphate binder types taken at baseline					
	Overall N = 204		One type N = 52		Two or more types N = 152	
	n (%)	95% CI	n (%)	95% CI	n (%)	95% CI
≥ 30% reduction	158 (77.5)	71.1, 83.0	38 (73.1)	59.0, 84.4	120 (78.9)	71.6, 85.1
Complete switching (100% reduction of phosphate binders)	93 (45.6)	38.6, 52.7	33 (63.5)	49.0, 76.4	60 (39.5)	31.6, 47.7

*CI* confidence interval.

From Week 0 to 52, the mean (SD) pill number of prescribed phosphate binders per day decreased from 11.4 (7.62) to 3.1 (5.48) (Fig. 3b). At Week 52, the mean (SD) change and mean (SD) percentage change from baseline were − 8.5 (5.60) pills per day and − 80.0% (25.5%), respectively, by switching from phosphate binders to tenapanor. The decrease in the mean number of daily pills is illustrated in Fig. 3c. The mean number of pills decreased for all individual phosphate binder types (Fig. 3d–i), with the most notable decreases observed for bixalomer and sevelamer. The total volume of prescribed phosphate binder and tenapanor pills decreased each week, with a mean (SD) percentage change from baseline of − 70.8% (25.3%) at Week 52.

The mean (SD) total weight of tenapanor pills prescribed daily at baseline was 102.0 (0.0) mg. Pill weight subsequently increased each week, reaching a total of 357.6 (214.6) mg at Week 52. For phosphate binders, the mean (SD) total pill weight prescribed daily at baseline was 5,730.8 (3,539.2) mg. The pill weight decreased each week, starting with the initial tenapanor treatment, finally reaching a total weight of 1,582.6 (2,679.4) mg at Week 52. The total combined weight of daily phosphate binder and tenapanor pills at Week 52 was 1,940.1 (2,737.7) mg. Mean (SD) change in weight from baseline was − 3,962.9 (2,777.2) mg; mean (SD) percentage change from baseline was − 71.9% (25.1%).

The mean (SD) total volume of tenapanor pills prescribed daily at baseline was 88.6 (0.0) mm^3^. Pill volume subsequently increased each week, reaching 296.8 (173.6) mm^3^ at Week 52. For phosphate binders, the mean (SD) total volume prescribed daily at baseline was 4,590.2 (3,525.1) mm^3^. The volume decreased each week, reaching 1,190.0 (2,173.1) mm^3^ at Week 52. The total combined volume of phosphate binder and tenapanor pills was 1,486.8 (2,208.0) mm^3^ at Week 52. Mean (SD) change in volume was − 3,186.4 (3,041.8) mm^3^; mean (SD) percentage change from baseline was − 70.8% (25.3%).

**Figure 4:**
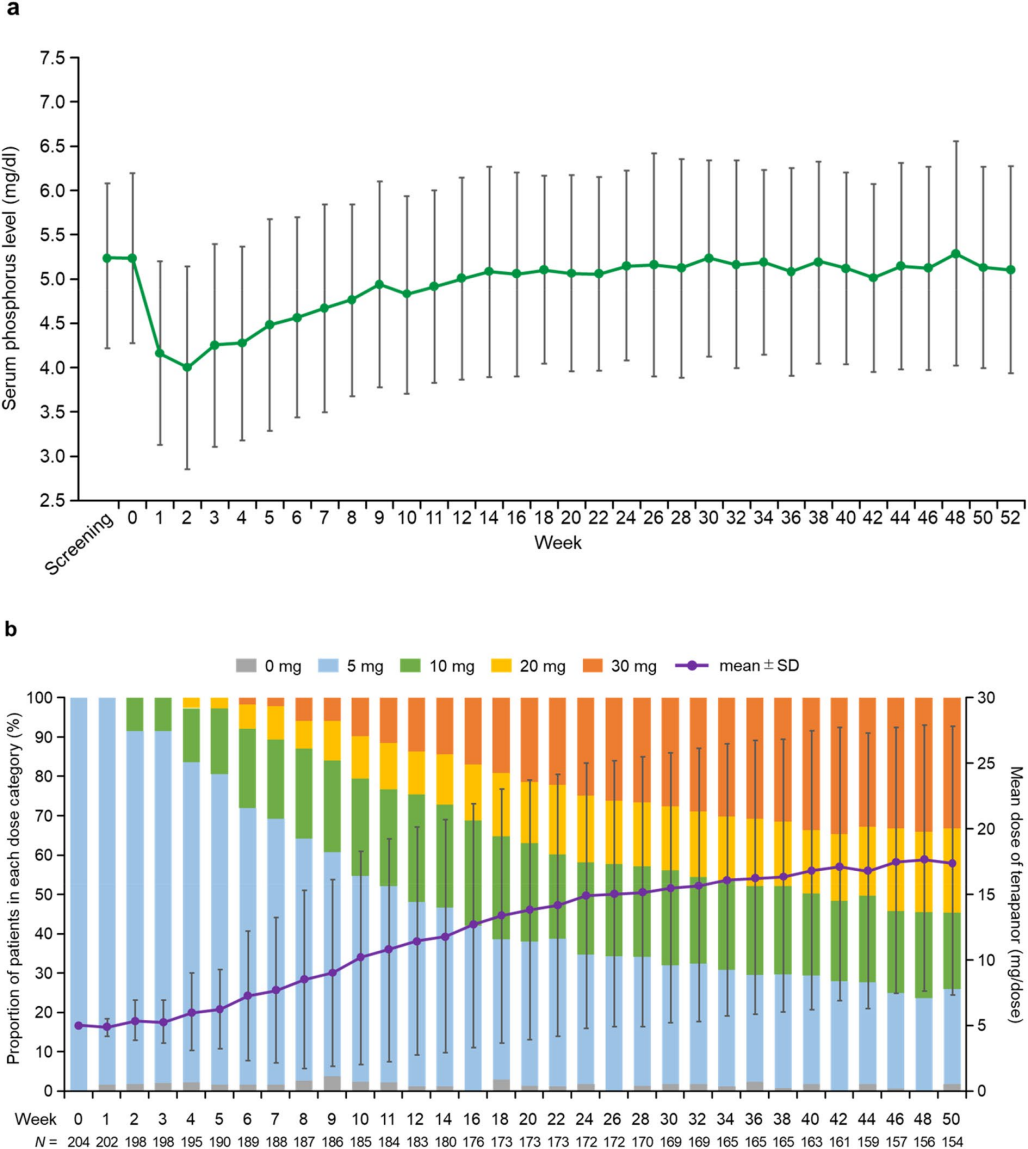
(**a**) Time course of mean serum phosphorus level; (**b**) proportion of patients in each dose category and mean dose of tenapanor during the study. SD, standard deviation.

Mean (SD) serum phosphorus levels decreased to 4.01 (1.14) mg/dl at Week 2, gradually increased until Week 10, and remained approximately stable until Week 52 and below baseline at all time points during the study (Fig. 4a). The mean (SD) serum phosphorus level at Week 52 was 5.11 (1.17) mg/dl, similar to baseline. While the pill number of phosphate binders was reduced for all individual phosphate binder types used at baseline, serum phosphorus levels remained well controlled throughout the study period for all types of phosphate binders (Supplementary Fig. S3). At Week 52, the proportion of patients receiving the 5, 10, 20, and 30 mg doses was 24.0%, 19.5%, 21.4%, and 33.1%, respectively. The tenapanor dose was steadily up-titrated throughout the study, reaching a mean dose of 17.4 mg at the end of the study (Fig. 4b).

## Discussion

After 52 weeks of tenapanor administration, the most common drug-related AE was diarrhea, occurring in approximately 55% of patients. The severity of diarrhea was classified as mild in 74.8%. The increase in Bristol Stool Form Scale score and stool frequency was similar to that reported in another Japanese phase 3 study^9^. No exacerbation of diarrhea was observed with long-term tenapanor treatment. Moreover, no drug-related AEs with a high incidence other than diarrhea were observed. Diverticular perforation and colitis occurred as drug-related SAEs. Although diverticular perforation and colitis have been observed in some nonclinical results, it is unclear if there is a mechanism to suggest causation with tenapanor. Tenapanor is a calcium-free, metal-free, non-polymeric agent that is poorly absorbed in the body. Therefore, the risk of tenapanor accumulation would be unchanged even with long-term treatment, possibly explaining the favorable findings regarding safety with long-term tenapanor administration in this study.

Bicarbonate levels decreased during 52-week administration in this study. Although there is a possible mechanism by which tenapanor might enhance chloride excretion by inhibiting NHE3 because sodium and hydrogen exchange is closely conjugated with chloride and bicarbonate exchange in the intestinal tract^13^, chloride levels did not change throughout the study. Thus, the mechanism for the decrease in bicarbonate levels is not clear.

Phosphate binders may be used while expecting secondary effects characteristic of each type, such as a decrease in total cholesterol and low-density lipoprotein cholesterol, replenishment of calcium, or iron supplementation. In this study, AEs associated with a decrease in phosphate binders were not observed. Similar to the findings reported in a 26-week phase 2 study^11^, given that no safety concerns were observed throughout long-term administration when adding tenapanor to phosphate binders and switching from phosphate binders to tenapanor, the current study suggests this switching approach may be safe and feasible in clinical practice.

Over 75% of patients achieved a ≥ 30% reduction in the total daily pill number of phosphate binders and tenapanor from baseline, which was the key secondary endpoint. Furthermore, the mean daily pill number of phosphate binders decreased from 11.4 to 3.1; thus, adding tenapanor reduced the mean number of pills taken by eight or more. Even when accounting for the addition of tenapanor (a daily dose of two tablets), the total number of pills per day was 5.1 at Week 52. Consequently, administration of tenapanor resulted in a ≥ 50% decrease in the mean daily pill burden of hyperphosphatemia treatment at Week 52 compared with baseline. Moreover, the mean daily volume of phosphate binders and tenapanor at Week 52 decreased by approximately 70% from baseline; significant reductions in both the volume and number of pills were noted. It is conceivable that the high serum phosphorus-lowering effect was a result of the additive effect associated with the difference between the mechanism of action of tenapanor^14^ and that of the existing phosphate binders. Additionally, approximately 45% of patients completely switched from phosphate binders during the study, reducing the pill burden on these patients. Given the numerous types of phosphate binders, they are often administered to patients in multiple combinations, thus potentially exacerbating problems of polypharmacy and worsening medication adherence. Although polypharmacy has a detrimental effect on health-related quality of life in dialysis patients^5,15–17^ eliminating phosphate binders by adding tenapanor may ameliorate these concerns substantially.

Even though the pill burden was reduced, mean serum phosphorus levels were well controlled within the target range (3.5–6.0 mg/dl) proposed by the Japanese Society for Dialysis Therapy^3^ throughout the study, and did not exceed the mean baseline level at any timepoint during the 52 weeks. These findings were consistently observed regardless of the phosphate binder used at baseline; tenapanor effectively lowered and then maintained serum phosphorus levels, irrespective of the type of phosphate binder used. While the mean daily number of pills decreased steadily early on for all phosphate binder types, serum phosphorus levels remained well controlled. It would be desirable to clarify whether managing serum phosphorus levels and decreasing phosphate binder pill burden can be observed in further long-term studies.

This study indicated a favorable effect on reducing phosphate binders regardless of the number of phosphate binder types taken at baseline. More than 70% of patients in this study received multiple types of phosphate binders at baseline. It is plausible that a similar effect on pill burden decrease could be shown in clinical practice, where many patients take multiple phosphate binders^18^. In addition, complete switching from phosphate binders was achieved in over 60% of patients who received a single phosphate binder type at baseline. It is conceivable that patients who received a single phosphate binder type could have a high potential to completely switch from phosphate binders to tenapanor compared with patients who received two or more phosphate binder types.

Some limitations of this study were, first, the single-arm, open-label design, meaning the findings were not validated against a placebo comparator. Second, only Japanese patients were included, precluding generalizability to other settings. Third, approximately 75% of patients took a combination of multiple phosphate binders at baseline. Phosphate binder dosing was adjusted according to the investigator’s judgment, which may have introduced bias regarding the types of phosphate binders to be reduced and by how many pills.

In conclusion, diarrhea was commonly reported, albeit cases tended to be mild in severity. No other safety issues were observed at a high incidence, suggesting long-term treatment safety and tolerability of tenapanor. In this population of patients whose serum phosphorus levels were controlled, many patients successfully reduced the use of phosphate binders by adding tenapanor. These findings suggest that, even in patients with refractory hyperphosphatemia and a high pill burden, tenapanor could potentially achieve the dual goals of reducing the pill number of phosphate binders and controlling serum phosphorus levels while contributing to ameliorating polypharmacy and medication adherence.

### Supplementary Information

Supplementary Figures.

Supplementary Text S1.

## Data Availability

The data underlying this article and generated during this study sponsored by Kyowa Kirin Co., Ltd. will be available in the Vivli repository https://vivli.org/ourmember/kyowa-kirin/ as long as conditions of data disclosure specified in the policy section of the Vivli website are satisfied. Go to https://vivli.org/members/enquiries-about-studies-not-listed-on-the-vivli-platform/ and fill out the form.
