# Supplementary figures and images for: Long-term safety and decrease of pill burden by tenapanor therapy: a phase 3 open-label study in hemodialysis patients with hyperphosphatemia
Source: Sci Rep. 2023 Nov 4;13:19100. doi: 10.1038/s41598-023-45080-9 (PMC10625594; doi:10.1038/s41598-023-45080-9)

## Slide 1
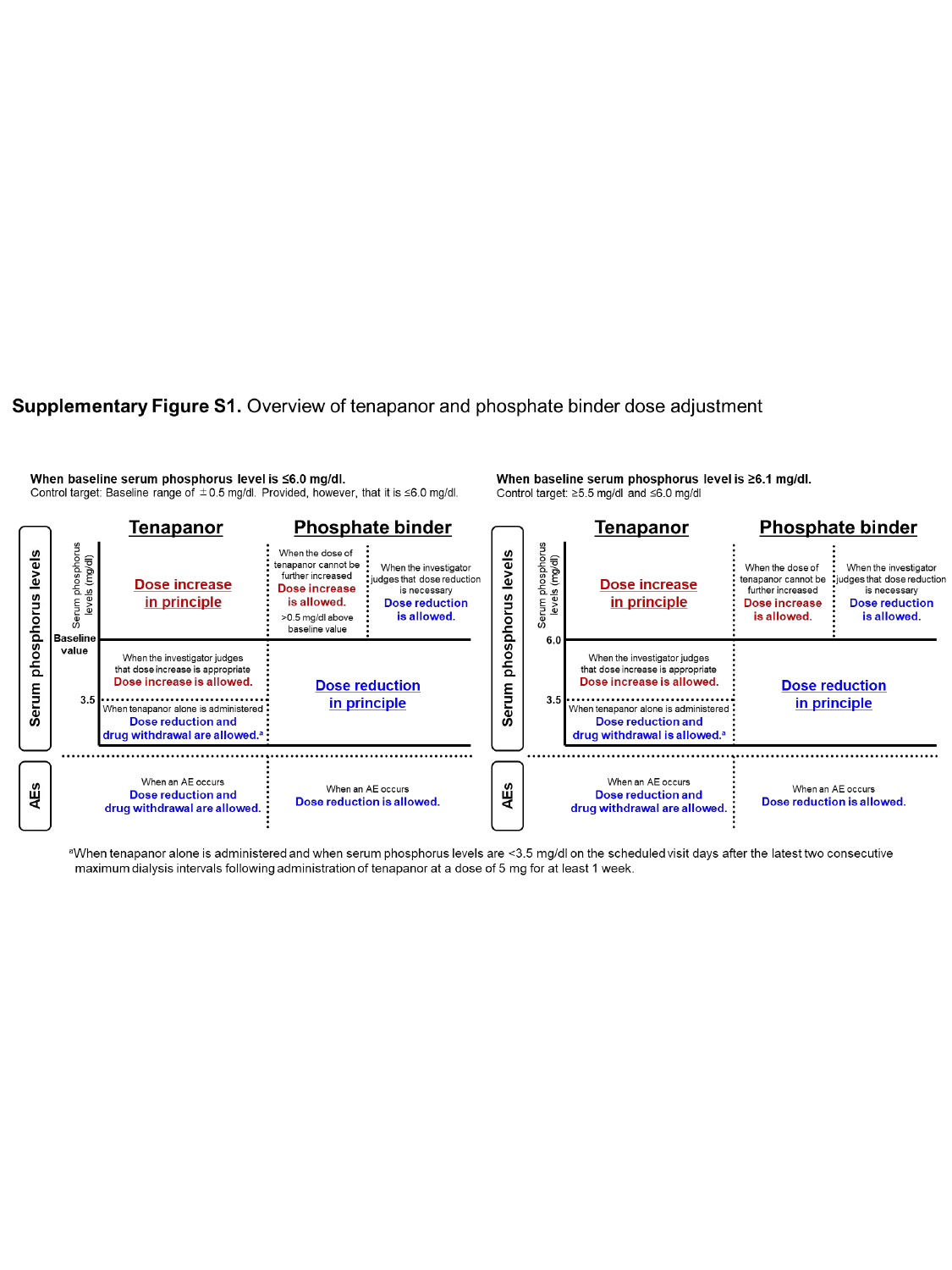

## Slide 2
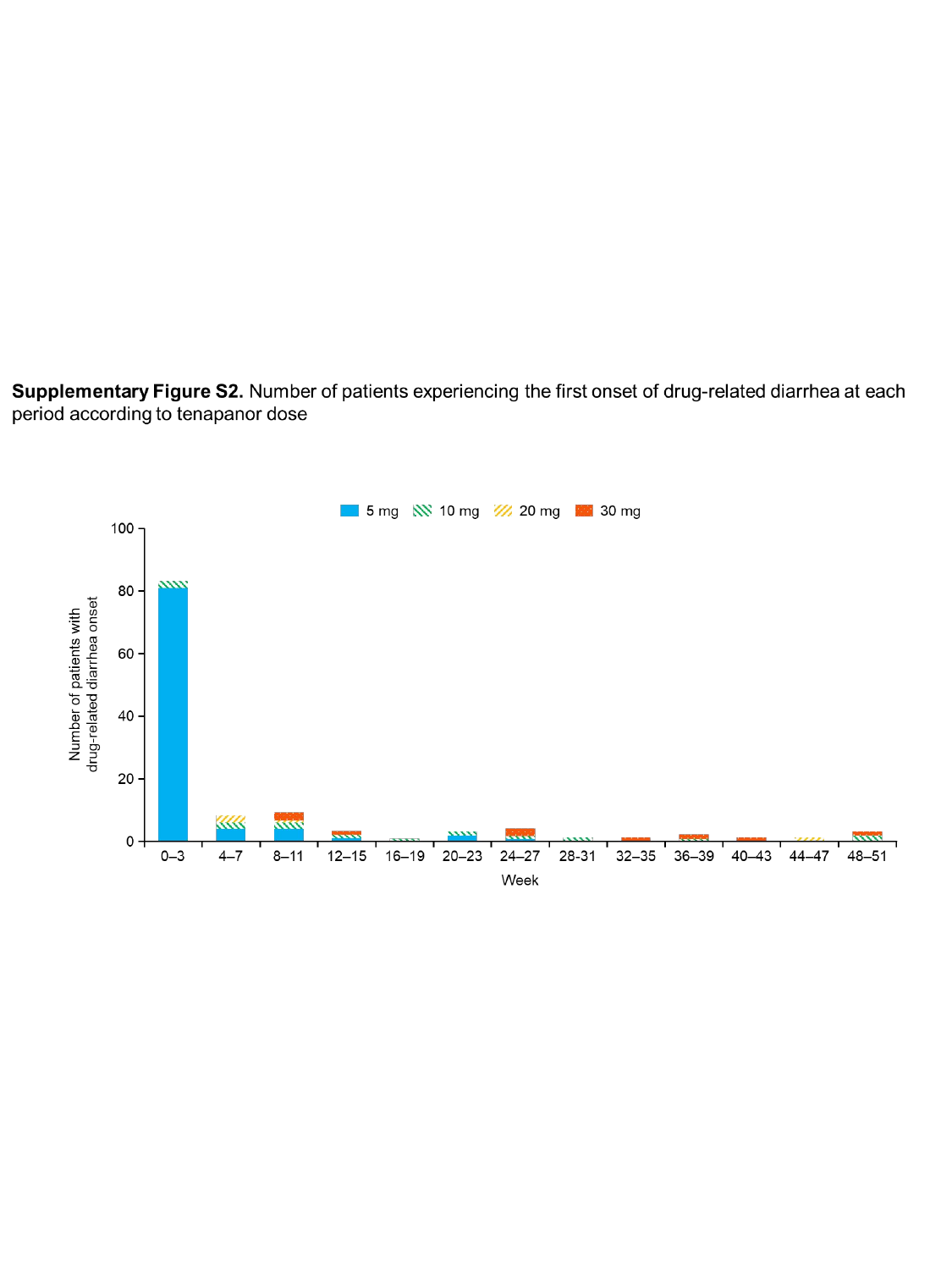

## Slide 3
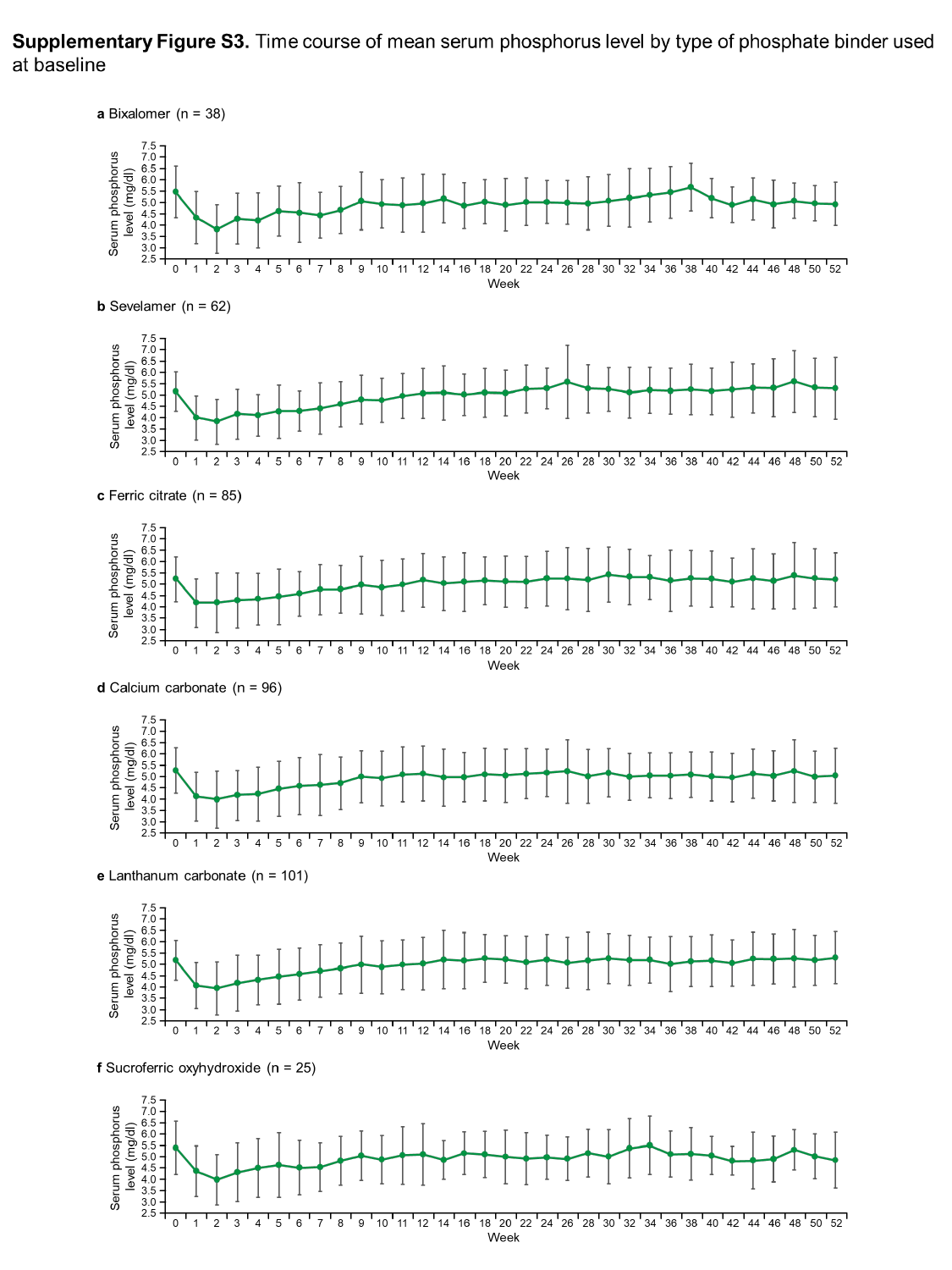

Supplement: Supplementary file 1 — Supplementary Figures. [file 41598_2023_45080_MOESM1_ESM.pptx]
