# Supplementary material for: Long-term safety and decrease of pill burden by tenapanor therapy: a phase 3 open-label study in hemodialysis patients with hyperphosphatemia
Source: Sci Rep. 2023 Nov 4;13:19100. doi: 10.1038/s41598-023-45080-9 (PMC10625594; doi:10.1038/s41598-023-45080-9)
Supplement: Supplementary file 2 — Supplementary Text S1. [file 41598_2023_45080_MOESM2_ESM.docx]

**Supplementary Material**

**Supplementary Figure S1.** Overview of tenapanor and phosphate binder dose adjustment

AE: adverse event

**Supplementary Figure S2.** Number of patients experiencing the first onset of drug-related diarrhea at each period according to tenapanor dose

**Supplementary Figure S3. a–f** Time course of mean serum phosphorus level by type of phosphate binder used at baseline

**Supplementary Text S1:** List of participating centers

| **Principal Investigator** | **Institution** | **Type of**  **Review Board** | **Name of External Review Board** |
| --- | --- | --- | --- |
| Kazutaka Kukita | Sapporo Hokuyu Hospital | External | Sapporo Medical Association's Institutional Review Board |
| Toko Endo | H. N. MEDIC | External | Review Board of Human Rights and Ethics for Clinical Studies Institutional Review Board |
| Kazuya Sakamoto | Tomakomai Nisshou Hospital | External | Review Board of Human Rights and Ethics for Clinical Studies Institutional Review Board |
| Masatsugu Sato | SATO Nephrology and Urology Clinic | External | Yokohama Minoru Clinic Institutional Review Board |
| Ikuto Masakane | Yabuki Hospital | External | Yokohama Minoru Clinic Institutional Review Board |
| Yoshitaka Maeda | JA Toride Medical Center | External | Review Board of Human Rights and Ethics for Clinical Studies Institutional Review Board |
| Sakae Ishii | Higashimatsuyama Kozin Clinic | External | Adachi Kyosai Hospital Institutional Review Board |
| Hidekazu Okamoto | Mashiko Hospital Dialysis Clinic | External | Adachi Kyosai Hospital Institutional Review Board |
| Takayuki Fujii | Seirei Sakura Citizen Hospital | External | Review Board of Human Rights and Ethics for Clinical Studies Institutional Review Board |
| Mitsuko Iwazaki | Toshiba Rinkan Hospital | External | Yokohama Minoru Clinic Institutional Review Board |
| Chie Ogawa | Maeda Institute of Musashikosugi Clinic | External | Jinbo Orthopedic Surgery Institutional Review Board |
| Kanji Shishido | Kawasaki Clinic | External | Yokohama Minoru Clinic Institutional Review Board |
| Hisaki Shimada | Shinrakuen Hospital | Internal |  |
| Hisanori Azekura | Sanaru Sun Clinic | External | Review Board of Human Rights and Ethics for Clinical Studies Institutional Review Board |
| Yosuke Saka | Kasugai Municipal Hospital | Internal |  |
| Masayoshi Yamaha | Daiyukai Daiichi Hospital | External | Review Board of Human Rights and Ethics for Clinical Studies Institutional Review Board |
| Yoshinari Tsuruta | Meiyo Clinic | External | Review Board of Human Rights and Ethics for Clinical Studies Institutional Review Board |
| Takeshi Onogi | Hekikai Kyoritsu Clinic | External | Review Board of Human Rights and Ethics for Clinical Studies Institutional Review Board |
| Kunihiro Nabeshima | Meiko Kyoritsu Clinic | External | Review Board of Human Rights and Ethics for Clinical Studies Institutional Review Board |
| Akikazu Yamamoto | Hakuyoukai Hospital | External | Review Board of Human Rights and Ethics for Clinical Studies Institutional Review Board |
| Masashi Yasutomi | Kuwana City Medical Center | External | Jinbo Orthopedic Surgery Institutional Review Board |
| Tsutomu Shikano | Kyoto Okamoto Memorial Hospital | External | Review Board of Human Rights and Ethics for Clinical Studies Institutional Review Board |
| Naoya Kodama | Kodama Hospital | External | Review Board of Human Rights and Ethics for Clinical Studies Institutional Review Board |
| Katsuhiko Arimoto | Shigei Hospital | External | Adachi Kyosai Hospital Institutional Review Board |
| Hiroaki Obayashi | Kinashi Obayashi Hospital | External | Yokohama Minoru Clinic Institutional Review Board |
| Takeaki Shinzato | Shinzato Clinic Urakami | External | Jinbo Orthopedic Surgery Institutional Review Board |
| Takuma Kojo | Ikeda Hospital | Internal |  |
| Yoshiteru Ohno | Ohno Memorial Hospital | External | Yokohama Minoru Clinic Institutional Review Board |
| Masatomo Taniguchi | Fukuoka Renal Clinic | External | Review Board of Human Rights and Ethics for Clinical Studies Institutional Review Board |
| Sunao Yamamoto | Sanin Rosai Hospital | Internal |  |
